# Exploring the interconnectedness between health-related quality of life factors among long-term adolescent and young adult cancer survivors (AYAs): a network analysis

**DOI:** 10.1007/s00520-023-08295-0

**Published:** 2024-01-13

**Authors:** Tom I. Bootsma, Deborah van de Wal, Carla Vlooswijk, Daniëlle C. Roos, Cas Drabbe, Renaud Tissier, Rhodé M. Bijlsma, Suzanne E.J. Kaal, Jan Martijn Kerst, Jacqueline M. Tromp, Monique E.M.M. Bos, Tom van der Hulle, Roy I. Lalisang, Janine Nuver, Mathilde C.M. Kouwenhoven, Winette T.A. van der Graaf, Silvie H.M. Janssen, Olga Husson

**Affiliations:** 1 Department of Medical Oncology, Netherlands Cancer Institute – Antoni van Leeuwenhoek, 1066 CX Amsterdam, The Netherlands; 2 Research and Development, Netherlands Comprehensive Cancer Organization, 3511 DT Utrecht, The Netherlands; 3 Department of Medical Oncology, Erasmus MC Cancer Institute, Erasmus University Medical Center, 3015 GD Rotterdam, The Netherlands; 4 Department of Psychosocial Research and Epidemiology, Netherlands Cancer Institute, 1066 CX Amsterdam, The Netherlands; 5 Department of Medical Oncology, University Medical Center Utrecht, 3584 CX Utrecht, The Netherlands; 6 Department of Medical Oncology, Radboud University Medical Center, 6525 GA Nijmegen, The Netherlands; 7 Department of Medical Oncology, Amsterdam University Medical Centers, 1105 AZ Amsterdam, The Netherlands; 8 Department of Medical Oncology, Leiden University Medical Center, 2333 ZA Leiden, The Netherlands; 9 Department of Internal Medicine, GROW-School of Oncology and Reproduction, Maastricht UMC+ Comprehensive Cancer Center, 6229 HX, Maastricht, The Netherlands; 10 Department of Medical Oncology, University Medical Center Groningen, 9713 GZ Groningen, The Netherlands; 11 Department of Neurology, Cancer Center Amsterdam, Brain Tumor Center, Amsterdam UMC, Amsterdam University Medical Centers, Location VUmc, 1081 HV Amsterdam, The Netherlands; 12 Department of Surgical Oncology, Erasmus MC Cancer Institute, Erasmus University Medical Center, 3015 GD Rotterdam, The Netherlands

**Keywords:** Adolescents and young adults, Cancer, Survivorship, Network analysis, HRQoL, Questionnaire study

## Abstract

**Purpose:**

Adolescent and young adult cancer survivors (AYAs) are at increased risk of long-term and late effects, and experience unmet needs, impacting their health-related quality of life (HRQoL). In order to provide and optimize supportive care and targeted interventions for this unique population, it is important to study HRQoL factors’ interconnectedness on a population level. Therefore, this network analysis was performed with the aim to explore the interconnectedness between HRQoL factors, in the analysis described as nodes, among long-term AYAs.

**Methods:**

This population-based cohort study used cross-sectional survey data of long-term AYAs, who were identified by the Netherlands Cancer Registry (NCR). Participants completed a one-time survey (SURVAYA study), including the EORTC survivorship questionnaire (QLQ-SURV111) to assess their long-term HRQoL outcomes and sociodemographic characteristics. The NCR provided the clinical data. Descriptive statistics and a network analysis, including network clustering, were performed.

**Results:**

In total, 3596 AYAs (on average 12.4 years post diagnosis) were included in our network analysis. The network was proven stable and reliable and, in total, four clusters were identified, including a worriment, daily functioning, psychological, and sexual cluster. Negative health outlook, part of the worriment cluster, was the node with the highest strength and its partial correlation with health distress was significantly different from all other partial correlations.

**Conclusion:**

This study shows the results of a stable and reliable network analysis based on HRQoL data of long-term AYAs, and identified nodes, correlations, and clusters that could be intervened on to improve the HRQoL outcomes of AYAs.

**Supplementary Information:**

The online version contains supplementary material available at 10.1007/s00520-023-08295-0.

## Introduction

Adolescent and young adult cancer survivors (AYAs), those aged 15–39 years at initial cancer diagnosis, are considered a unique group with age-specific challenges, from cancer diagnosis until end of life [1, 2]. This age range is, however, flexibly applied depending on the research question of interest, country, and health care system [3]. In the Netherlands, pediatric oncology is centralized and includes patients until 18 years of age at diagnosis. The Dutch AYA definition applies to all cancer patients initially diagnosed between 18 and 39 years. As the overall cancer incidence of AYAs has increased over the last decades and the 5-year relative survival is now exceeding 80%, a large part of this growing population will eventually become long-term survivors [4]. As a result of the cancer diagnosis and treatment, these AYAs are at increased risk of long-term (e.g., infertility) and late effects (e.g., secondary malignancies), and experience unmet (age-specific) needs related to finances and mental health for example [5–7]. AYAs are in a particularly exposed position for these risk factors due to their often invasive and long-lasting treatments [8], and being diagnosed during a complex phase of life, including many physical, emotional, and social transitions [9].

AYAs can have a long life ahead in which suffering from these long-term and late effects can have a significant impact on their health-related quality of life (HRQoL). HRQoL is defined by the survivor’s own perception of one’s health or well-being, including physical, mental, and social aspects [10]. Several studies have focused on the HRQoL issues among AYAs: literature shows that AYAs are at increased risk of fatigue [11], cognitive impairment [12], work and financial problems [12, 13], psychological distress [14], and body image issues [15], which can result in a diminished HRQoL. In addition, studies described impacted physical health and functioning [16–19], and lower mental health [17–19] in AYAs compared to the general population/older cancer survivors [19]. In line, the systematic review of Quinn showed that AYAs are more likely to have impaired HRQoL compared to the general population, although QoL was difficult to measure due to their age-specific needs [20].

Many factors can independently impact HRQoL, yet they often co-occur in cancer patients [21–25]. To study the interconnectedness between these factors, a network analysis can be performed. It provides insight into the relationships among symptoms, risk factors, and protective factors. Network approaches involve the identification of symptoms and factors (network nodes) and the relations among them (positive or negative associations between nodes) [26]. Taking into account the dependence of factors, this type of analysis is more likely reflecting reality compared to focusing on these factors independently [27].

Although network analyses have been performed previously among cancer survivors in general [27–29], they are lacking among AYAs, especially when focusing on HRQoL outcomes. Gaining these insights is of importance to optimize supportive care and provide targeted interventions for this unique long-term surviving population. Within a large population-based sample of long-term AYAs, using an exploratory approach, we want to (1) assess HRQoL in long-term AYAs, (2) identify the most central nodes in a HRQoL network, and (3) determine how these nodes are linked to one another ((strengths of) interconnectedness).

## Methods

### Study population and data collection

Data of the population-based, cross-sectional SURVAYA study was used, which was approved by the Institutional Review Board (IRBd18122) and registered within clinical trial registration (NCT05379387). The study population is extensively described previously [30]. In short, the SURVAYA study was performed among AYAs (18–39 years old at time of initial cancer diagnosis) diagnosed with cancer between 1999 and 2015 (5–20 years post diagnosis at study invitation), treated at the Netherlands Cancer Institute or one of the University Medical Centers in the Netherlands, and registered within the Netherlands Cancer Registry (NCR). Survivors were invited to complete a one-time questionnaire within PROFILES (Patient Reported Outcomes Following Initial treatment and Long-term Evaluation of Survivorship) [31].

### Measures

Sociodemographic characteristics were obtained through a one-time questionnaire including age at time of questionnaire, sex at birth, marital status, and educational level. Clinical characteristics, obtained by the NCR, include tumor type, stage, primary treatment received, and time since diagnosis. Tumor type was classified according to the third International Classification of Diseases for Oncology (ICDO-3) [32]. Cancer stage was classified according to TNM or Ann Arbor Code (Hodgkin lymphoma and Non-Hodgkin lymphoma) [33].

The EORTC QLQ-SURV111 [34], a cancer core survivorship questionnaire that is currently being developed by the European Organization for Research and Treatment of Cancer (EORTC), was used for our network analysis. This questionnaire assesses long-term HRQoL outcomes, including physical, mental, and social HRQoL issues specifically relevant to cancer survivors. We selected 8 functioning scales (physical functioning, cognitive functioning, emotional functioning, role functioning, body image, symptom awareness, sexual functioning, and overall quality of life), 9 symptoms scales (fatigue, sleep problems, pain, social interference, health distress, negative health outlook, social isolation, symptom checklist, and sexual problems), and 3 single items (financial difficulties, worry cancer risk family, and treated differently). Scales and items measuring positive factors of HRQoL and items that were not applicable for all participants were excluded from the analysis. The rationale for this selection is that including these positive scales/items and optional items would result in difficulties with respectively interpreting network associations to intervene on and the development of a network structure. Participants scored the items on a 4-point Likert scale from 1 (not at all) to 4 (very much). Overall quality of life scores ranged from 1 (very poor) to 7 (excellent). All scales and single items were linearly transformed to a “0–100” scale [35]. A higher score on the functioning scales indicates better HRQoL/functioning, while a higher score for symptoms indicates more complaints. For our analysis, we transformed the scores of the symptom scales once again, so a higher score on the symptom scale indicates fewer complaints. Now, a higher score on all scales corresponds with better functioning, fewer complaints, and a better overall quality of life.

### Data analysis

Data were analyzed using SPSS Statistics (IBM Corporation, version 26.0, Armonk, NY, USA) and R version 4.2.1 packages MVN, huge, qgraph, and bootnet. Descriptive statistics were calculated and presented as frequencies, percentages, means, and standard deviations.

We used listwise deletion to exclude participants with missing items on the EORTC QLQ-SURV111 questionnaire, except for the items related to sexuality. This is because sexual functioning and problems can be an important aspect of HRQoL, and missing responses to sensitive questions like sexuality are common [36]. If half of the items from the two sexuality scales were answered, we assumed that the missing item had a value equal to the item that was present for that respondent according to the QLQ-SURV111 scoring manual. In case both items for the scale were missing, we used a copy mean imputation of the study population.

We assessed the assumption of multivariate normality with Mardia’s test [37], which needs to be fulfilled prior to estimating the network [38]. Mardia’s multivariate skewness and kurtosis coefficients of the numeric scales were calculated [37]. In the case of multivariate normality, both *p*-values of skewness and kurtosis should be greater than 0.05 [37]. As the data were not multivariate normally distributed according to Mardia’s test (*p*<0.05), a nonparanormal transformation was applied to relax the normality assumption [39].

In our network model, HRQoL is conceptualized as a network of mutually interrelated factors. Because data are continuous scales or items, we used Guassian Graphical model (GGM) [26, 40]. Nodes represent the selected HRQoL scales and items, and edges (links connecting two nodes) represent the regularized partial correlation coefficients after controlling for all other nodes. The thickness of the edge visualizes the strength, and the color a positive (red) or negative (blue) partial correlation. The partial correlation is indicated as very small (*r*<0.1), small (0.1≤*r*<0.3), moderate (0.3≤*r*<0.5), and large (*r*>0.5) [41].

We applied graphical lasso tuned with the Extended Bayesian Information Criterion (EBIC) [38, 42]. The EBIC hyperparameter, used to set the preferred simplicity of the model, was set to 0.5 to minimize spurious connections [38]. Graphical lasso is a form of lasso regularization to prevent that edges between two nodes are spurious because of other nodes (i.e., conditional independence association) and small edges were shrinked to zero by dropping them from the model [42]. In this way, the estimated network is not over fitted and interpretable.

We estimated node strength (i.e., number and strength of edges between nodes), betweenness (i.e., how often a node lies in shortest path between any combination of two nodes), and closeness (i.e., average distance from one node to all other nodes, which indicates how fast a node can be reached), which are indices of node centrality [38, 43].

Bootstrapping was performed to explore the accuracy and stability of the network [38]. To estimate the accuracy of edge weights, 95% bootstrapped confidence intervals (CIs) around each edge in the network were calculated. Non-parametric bootstrapping (1000 bootstrap samples) was used to construct CIs. To estimate the stability of node centrality, we applied case-dropping bootstrap (1000 bootstrap samples) to calculate the correlational stability coefficient (CS coefficient) [38]. This coefficient represents the maximum proportion of participants that can be dropped from the analysis with the correlation between the original centrality indices and the subset centrality indices of at least 0.7 with 95% probability [44]. The CS coefficients of at least above 0.25, but preferable above 0.5, are considered stable [44]. Additionally, the bootstrapped values were used to test the significance of edge weights and node strength [38]. These bootstrapped difference tests indicate the difference between two different edge weights or node strengths. A bootstrapped CI around these difference scores was calculated [38].

Detection of communities was performed using the Louvain clustering method, a hierarchical clustering method based on multi-level modularity optimization algorithm [45].

## Results

### Characteristics AYA cancer survivors

In total, 11296 AYAs were invited to participate in the study, of whom 4010 (36%) responded. After excluding 414 records with missing data, we included 3596 AYAs in our final analysis; their sociodemographic and clinical characteristics are described in Table 1. AYAs were on average 31.5 years old at diagnosis and mostly female (61%). The average time since diagnosis was 12.4 years and the most common cancer types were breast cancer (24%), germ cell tumors (18%), lymphoid hematological malignancies (15%), and tumors of female genitalia (11%).

**Table 1:** Sociodemographic and clinical characteristics of the study cohort

		Respondents N = 3596	
		*n*	%
Gender	Male	1420	39
	Female	2176	61
Age at diagnosis mean (SD)		31.5 (5.9)	
	18–24 years	569	16
	25–34 years	1584	44
	35–39 years	1443	40
Age at completing questionnaire mean (SD)		44.5 (7.5)	
Time since diagnosis		12.4 (4.5)	
	5–10 years	1452	40
	11–15 years	1247	35
	16–20 years	897	25
Type of cancer	Breast cancer	846	23.5
	Germ cell tumors	637	17.7
	Lymphoid hematological malignancies	540	15.0
	Female genitalia tumors	383	10.7
	Melanoma	252	7.0
	Thyroid cancer	224	6.2
	Bone or soft tissue sarcoma	161	4.5
	Myeloid hematological malignancies	136	3.8
	Central nervous system tumors	131	3.6
	Head and neck cancer	107	3.0
	Digestive tract tumors	104	2.9
	Other*	75	2.1
Tumor stage	I	1537	42.7
	II	957	26.6
	III	515	14.3
	IV	165	4.6
	Missing	422	11.7
Primary treatment modality	Surgery	2799	77.8
	Chemotherapy	2030	56.5
	Radiotherapy	1716	47.7
	Hormonal therapy	435	12.1
	Targeted therapy	282	7.8
	Stem cell therapy	130	3.6
Marital status (at time of questionnaire)	In a relation	3001	83.5
Educational level	Primary school or equivalent	16	0.4
	Secondary school or equivalent	1514	42.1
	College/university or equivalent	2060	57.3
	Missing	6	0.2

*Other includes urinary tract, respiratory tract, male genitalia, neuroblastoma, adrenal, paraganglioma, and eyes

The mean scores of the functioning and symptom scales of the QLQ-SURV111 are shown in Table 2. The overall global quality of life score of AYAs was on average 77.3 (SD 18.7). The functioning scale with the highest score was physical functioning (mean 91.7; SD 13.9), whereas sexual functioning was the scale with the lowest score (mean 43.7; SD 25.5). AYAs scored the lowest on the symptom scales social isolation (mean 69.1; SD 30.7), fatigue (mean 70.3; SD 26.0), and negative health outlook (mean 74.2; SD 20.0).

**Table 2:** The long-term HRQoL outcomes from the cancer survivorship core questionnaire (QLQ-SURV111) of the AYA cancer survivors, arranged by scale and total score mean

Scales	Short names	Functional/symptom	Number of items (*n*)	Item numbers	Raw scores mean (SD)	Total scores mean (SD)
Physical functioning	PF	Functional	5	1–5	1.2 (0.4)	91.7 (13.9)
Role functioning	RF	Functional	3	69–71	1.5 (0.7)	83.7 (24.5)
Emotional functioning	EF	Functional	7	52–58	1.6 (0.6)	81.0 (19.9)
Cognitive functioning	CF	Functional	4	47, 48, 50, 51	1.6 (0.7)	79.6 (22.6)
Body image	BI	Functional	2	40, 41	1.7 (0.7)	77.8 (24.6)
Overall quality of life	QL	Functional	1	121	5.6 (1.1)	77.3 (18.7)
Symptom awareness	SA	Functional	2	76, 77	2.2 (0.8)	60.9 (25.4)
Sexual functioning	SF	Functional	2	111, 112	2.3 (0.8)	43.7 (25.5)
Financial difficulties	FD	Symptom	1	68	1.3 (0.7)	89.4 (23.8)
Social interference	Sif	Symptom	2	73, 74	1.4 (0.7)	88.1 (22.0)
Worry cancer risk family	WF	Symptom	1	61	1.4 (0.7)	86.6 (23.1)
Symptom checklist	SC	Symptom	17	20, 21, 23–26, 28, 30–38, 75	1.4 (0.4)	85.8 (13.5)
Treated differently	TD	Symptom	1	107	1.4 (0.7)	85.2 (22.6)
Pain	PA	Symptom	2	22, 72	1.5 (0.7)	84.2 (23.0)
Sexual problems	SP	Symptom	2	113, 117	1.5 (0.8)	82.8 (26.3)
Health distress	HD	Symptom	3	63–65	1.6 (0.7)	79.0 (22.3)
Sleep problems	SL	Symptom	4	16–19	1.8 (0.7)	74.5 (23.6)
Negative health outlook	NHO	Symptom	7	60, 62, 64, 81, 82, 98, 99	1.8 (0.6)	74.2 (20.0)
Fatigue	FA	Symptom	4	6–9	1.9 (0.8)	70.3 (26.0)
Social isolation	SI	Symptom	2	89, 90	1.9 (0.9)	69.1 (30.7)

### Network analysis

#### Overall network

**Fig. 1:**
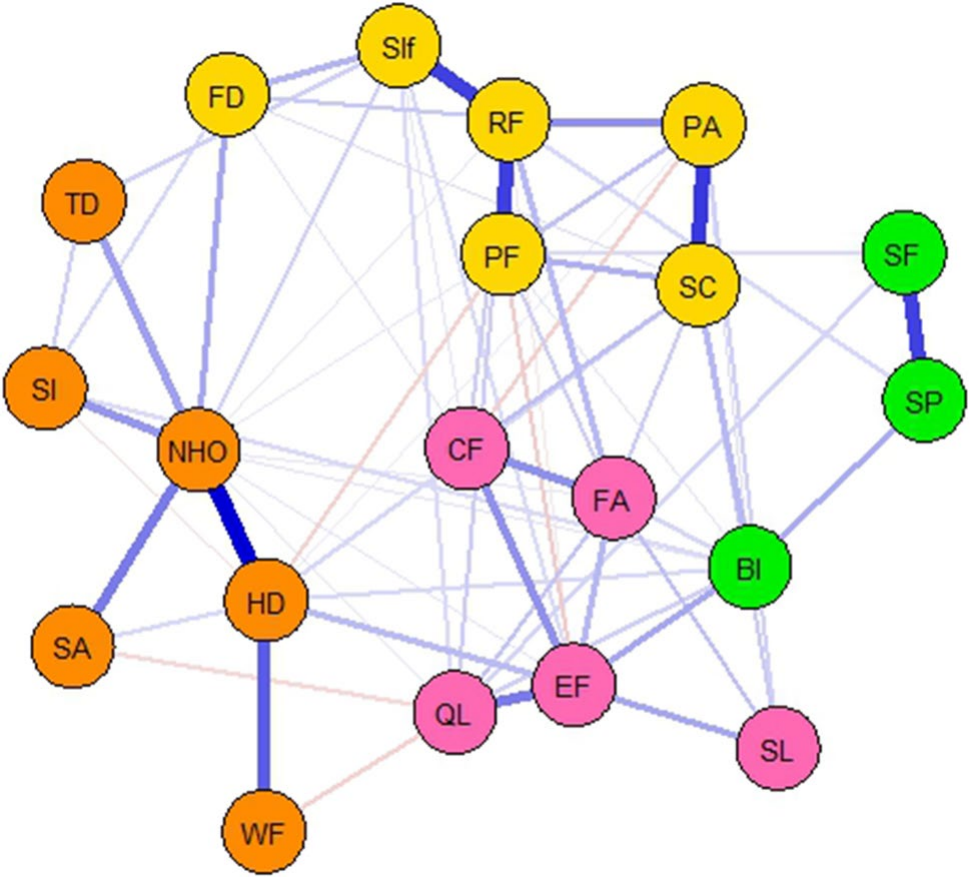
The (cluster) network of HRQoL outcomes of long-term AYA cancer survivors. In this partial correlation network model, the nodes (PF: physical functioning, CF: cognitive functioning, EF: emotional functioning, RF: role functioning, BI: body image, SA: symptom awareness, SF: sexual functioning, FA: fatigue, SL: sleep problems, PA: pain, Sif: social interference, HD: health distress, NHO: negative health outlook, SI: social isolation, SC: symptom checklist, SP: sexual problems, FD: financial difficulties, WF: worried about family getting cancer, TD: people treating you differently, QL: overall quality of life) represent all the HRQoL scales of the QLQ-SURV111 and the edges (links connecting two nodes) represent the regularized partial correlation coefficients after controlling for all other nodes. The blue color indicates a positive partial correlation and a red color a negative partial correlation between two nodes. The thickness of the edge visualizes the strength of the partial correlation between two nodes. The four clusters that we identified within our network model include in orange the worriment cluster, in yellow the daily functioning cluster, in pink the psychological cluster, and in green the sexual cluster

The partial correlation network model is shown in Fig. 1. In our sample, health distress had a strong partial correlation with negative health outlook (*r* = 0.71) and moderate partial correlation with worries about family getting cancer (*r* =0.48). Symptom checklist had a strong partial correlation with pain (*r* =0.67). Role functioning was strongly partially correlated to physical functioning (*r* =0.70) and to social interference (*r* =0.72), and there was a moderate partial correlation between sexual functioning and sexual problems (*r* =0.43).

**Fig. 2:**
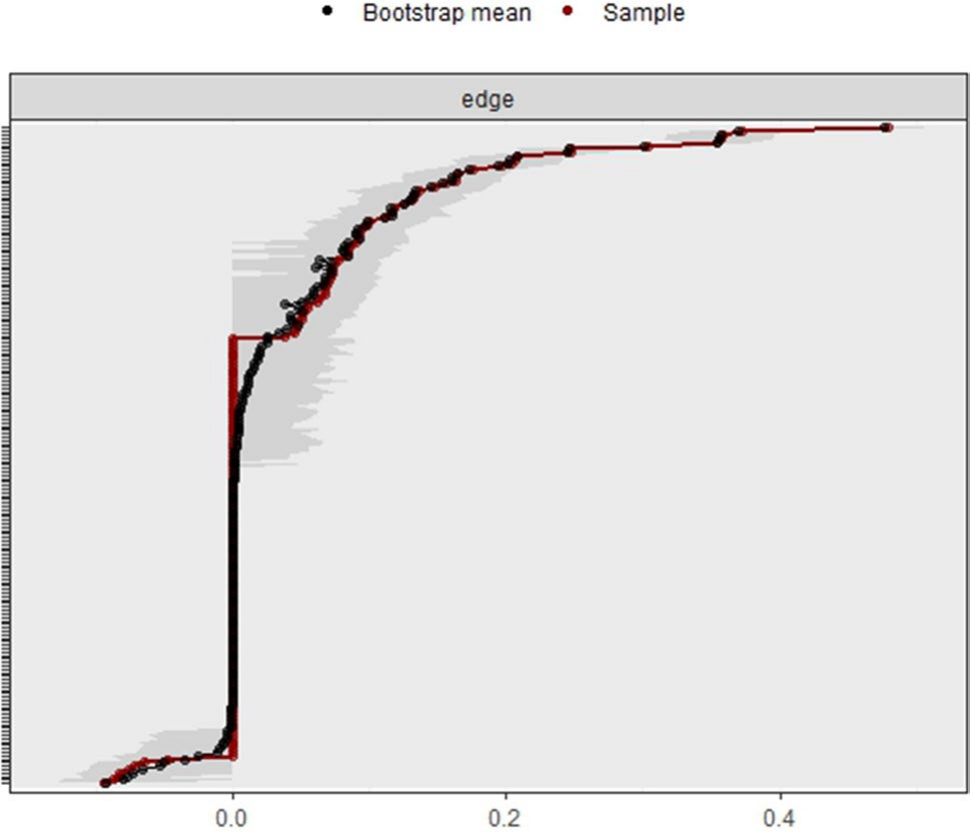
Bootstrapped confidence intervals of estimated edge weights. Each horizontal line represents one edge of the network, ordered from the edge with the highest edge weight to the edge with the lowest edge weight. The red line indicates the sample values and the gray lines are the bootstrapped CIs. The larger the gray line the less certain the edge value is

The bootstrapped confidence intervals of estimated edge weights (Fig. 2) show that the previously described strong/moderate correlations in our network are robust. The negative correlations in our network, on the other hand, are not reliable. To test if a correlation between two nodes was significantly different from other correlations, we used the edge difference test (Supplementary material Figure S1). This plot showed that the correlation between health distress and negative health outlook was significantly different from all other correlations.

#### Cluster analysis

Within our network model, we identified four clusters (Fig. 1); (1) the worriment cluster (orange), (2) the daily functioning cluster (yellow), (3) the psychological cluster (pink), and (4) sexual cluster (green). The worriment cluster consists of the nodes: health distress, negative health outlook, symptom awareness, social isolation, worried about family getting cancer, and people treating you differently. Role functioning, physical functioning, pain, symptom checklist, social interference, and financial difficulties were all part of the daily functioning cluster. Emotional functioning, fatigue, sleep problems, cognitive functioning, and overall quality of life formed the psychological cluster. The sexual cluster consists of sexual functioning, sexual problems, and body image.

#### Network stability and centrality

**Fig. 3:**
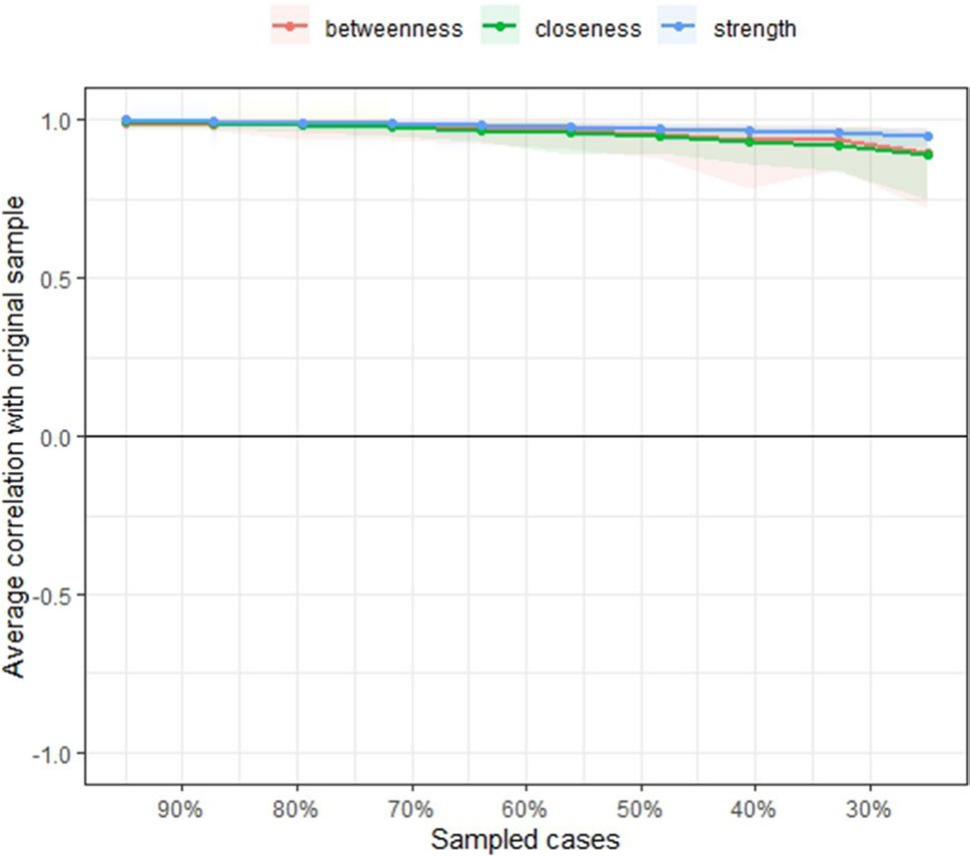
Correlational stability plot of centrality indices by case-dropping subset bootstrap. Correlations between centrality indices of network sampled with persons dropped and the original sample. Lines indicate the means and areas indicate the range from the 2.5th quantile to the 97.5th quantile

**Fig. 4:**
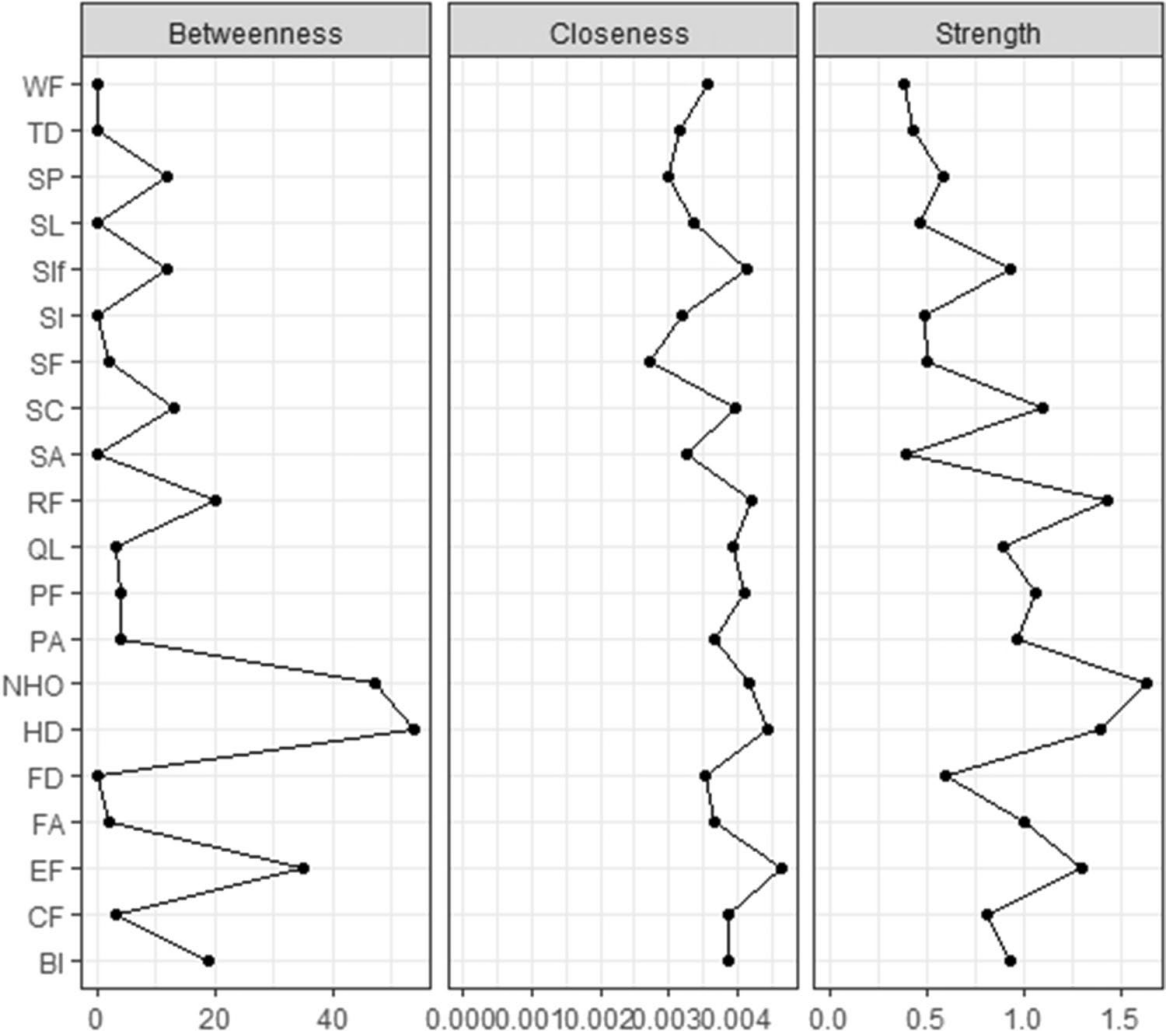
Central indices of the network. Centrality indices are shown as standardized *z*-scores. PF: physical functioning, CF: cognitive functioning, EF: emotional functioning, RF: role functioning, BI: body image, SA: symptom awareness, SF: sexual functioning, FA: fatigue, SL: sleep problems, PA: pain, Sif: social interference, HD: health distress, NHO: negative health outlook, SI: social isolation, SC: symptom checklist, SP: sexual problems, FD: financial difficulties, WF: worried about family getting cancer, TD: people treating you differently, QL: overall quality of life

The CS coefficient for strength, closeness, and betweenness were 0.75, 0.75, and 0.75 respectively, indicating a stable and reliable network (Fig. 3). Regarding centrality, the nodes with the highest strength are the most central, and therefore the most important nodes of the network model. In our model, nodes with the highest strength were negative health outlook (standardized centrality estimates (SCE) = 1.60), role functioning (SCE = 1.40), health distress (SCE = 1.40), and emotional functioning (SCE = 1.30). In addition, health distress and negative health outlook had the highest betweenness, and emotional functioning and health distress had the highest closeness (Fig. 4). The centrality difference plot (Supplementary material Figure S2) demonstrates that the strength of the node negative health outlook significantly differed from the other nodes in the network model.

## Discussion

This study shows the results of a stable and reliable network analysis based on HRQoL data of 3596 long-term AYAs, as the first to our knowledge. Although the overall global quality of life score was 77.3 on average, the lowest functioning scale score was sexual functioning and the lowest symptom scale scores were social isolation, fatigue, and negative health outlook. This network showed several strong/moderate partial correlations, with the partial correlation between health distress and negative health outlook being the only one significantly different from all other. Also, the strength of the node negative health outlook was significantly different from all other nodes. In total, four clusters of negative symptom and functioning HRQoL scales were identified, including a worriment cluster, daily functioning cluster, psychological cluster, and sexual cluster.

As this study is the first to apply a network analysis based on a wide range of HRQoL data of AYAs using the relatively new QLQ-SURV111 questionnaire, findings are difficult to compare with other studies. Although previous studies had similar aims, they mostly used the EORTC QLQ-C30 to assess HRQoL and studied adult cancer survivors [28, 46]. This was also the case in the network analysis conducted by Rooij et al. in a heterogeneous sample of adult cancer survivors where the EORTC QLQ-C30 was used [28]. In their analysis, fatigue was consistently central and had moderate direct relationships with emotional symptoms, cognitive symptoms, appetite loss, dyspnea, and pain. Fatigue being the most central symptom was in contrast with our results, which might be explained by the much younger population in our study (31.5 years vs 61 years) and the difference in time since diagnosis, which was considerably longer in our study (12.4 years vs 4.2 years). In our network, negative health outlook was the node with the highest strength, which had a strong correlation with health distress within the worriment cluster. This suggests that psychological and emotional issues remain more of relevance to AYA cancer survivors also after long-term follow-up, and are better picked up by the QLQ-SURV111 questionnaire that covers a more complete range of relevant survivorship issues.

Other network studies focused specifically on a construct (e.g., fear of recurrence) or predefined clusters of symptoms, for example, the network analysis on fear of cancer recurrence, anxiety, and depression in breast cancer patients of Yang et al. [46]. In their network, “having trouble relaxing” was the most central node, anxiety and depression were well-connected, and fear of cancer recurrence formed a distinct cluster. The use of the broad range of survivorship issues of the QLQ-SURV111, including psychological, social, physical symptoms and functioning, allowed us to explore clusters within our network as well. An interesting cluster that emerged was the worriment cluster, which consisted of negative health outlook, health distress, symptom awareness, social isolation, worried about family getting cancer, and people treating you differently. Although our questions regarding worries were different, one other network analysis on AYAs focused on the construct of fear of cancer recurrence [47]. Here, the researchers found fear of serious medical interventions as the most central symptom in their network, with the highest node strengths for fear of pain, fear of relying on strangers for activities of daily living, and fear of severe medical treatments. Based on their results, which emphasize the centrality of emotional issues among AYA patients, they stress the importance of prioritizing these symptoms for interventions. However, as stated previously, comparing the results of these studies with our results should be done cautiously as study populations, study designs, aims, and used questionnaires differ. The lack of studies among AYAs to compare our findings with stresses the need for more AYA-specialized research.

The results of a network analysis can provide more insight in the HRQoL of AYAs, in which symptoms and functioning can influence each other, instead of perceiving them as individual factors [48]. Identifying the most important factors in a network can help to address these problems with targeted interventions and healthcare, and lead to novel research ideas. First, we recommend to replicate network analysis studies in other groups of AYAs to be able to make comparisons between studies with similar study populations and draw conclusions with more certainty—changing the exploratory approach into a confirmative approach. Ideally, longitudinal data needs to be collected to draw conclusions over time and adapt healthcare to these time-related changes where needed. This could even be specific to longitudinal ecological momentary assessment (EMA) in which participants are asked to complete (parts of) the expected momentary dynamic items of the HRQoL questionnaire multiple times a day during a study period. In this way, variations over time can be taken into account to a more detailed and individual level. Inter- and intra-individual differences over time might result in changes in prevalence (scores of the scales or items), partial correlations of nodes, centrality, and clusters formed.

In addition, subgroup analysis should be performed to make healthcare interventions even more tailored. AYAs subgroup analysis could focus on age, gender, stage, treatment, and type of cancer. In order to establish tailored care, it is important to make the subgroups as specific as possible while remaining a stable and reliable network. However, it should also be noted that these results represent means and thus differences may exist on an individual level in clinical practice.

Future research can lead to and optimize supportive care and targeted interventions, like psycho-oncological care and psychosocial, behavioral, and supportive interventions [49]. For example, the strong significant correlation between health distress and negative health outlook, which is part of the worriment cluster in this study, might be intervened on by distress screening and renewed/tailored, age-specific psycho-oncological aftercare [50]. For healthcare providers (HCPs) involved in AYA healthcare, and in general, visualization of the nodes, correlations, and clusters may help to understand the cohesion between different factors/symptoms and the influence they might have on each other, and more important, how a targeted intervention can influence several HRQoL outcomes simultaneously. This advocates for holistic and age-specific psycho-oncological aftercare in which multiple factors are targeted simultaneously by a multidisciplinary team of HCPs, to be as efficient and effective as possible.

## Strengths and limitations

This explorative study represents the very first network analysis using data on a range of HRQoL outcomes of long-term AYAs to our knowledge. Strengths include the large sample size, the establishment of a stable and reliable network, and the inclusion of a wide range of survivorship issues. However, the results should be interpreted with caution as the EORTC QLQ-SURV111 is not yet finalized and validated. In addition, our study included mostly females and over 40% was diagnosed with a stage I tumor. With a response rate of 36%, there are several subgroups (males, AYAs with a more aggressive disease, and AYAs diagnosed at the age of 18–24) underrepresented in this analysis who might have different HRQoL outcomes [30]. Results may therefore not be generalizable to the total AYAs population. Also, we have not taken a closer look at the outcomes of specific subgroups (between groups), as the study group as a whole was analyzed. As mentioned previously, the subgroup analyses should be part of future research to tailor interventions with a risk-based approach. In line with this, due to the methodology of this study, i.e., a cross-sectional questionnaire study, no causal pathways or changes in HRQoL factors over time can be assessed. In the future, this might be tackled by using longitudinal data instead of cross-sectional data.

## Conclusions

This innovative network analysis provides insight in the nodes, correlations, and clusters that could be targeted to improve the HRQoL outcomes of AYAs. Future studies with longitudinal data and subgroup analyses can tailor the interventions and provided healthcare even more, specifically for those at risk of poor HRQoL outcomes. With these insights, more targeted interventions and healthcare can be provided and developed.

### Supplementary information

ESM 1 (DOCX 98 kb)

## Data Availability

The data presented in this study are available on request from the corresponding author. The data are not publicly available due to privacy issues.
