# Supplementary material for: Exploring the interconnectedness between health-related quality of life factors among long-term adolescent and young adult cancer survivors (AYAs): a network analysis
Source: Support Care Cancer. 2024 Jan 13;32(2):104. doi: 10.1007/s00520-023-08295-0 (PMC10787889; doi:10.1007/s00520-023-08295-0)
Supplement: Supplementary file 1 — (DOCX 98 kb) [file 520_2023_8295_MOESM1_ESM.docx]

**SUPPLEMENTARY MATERIALS**


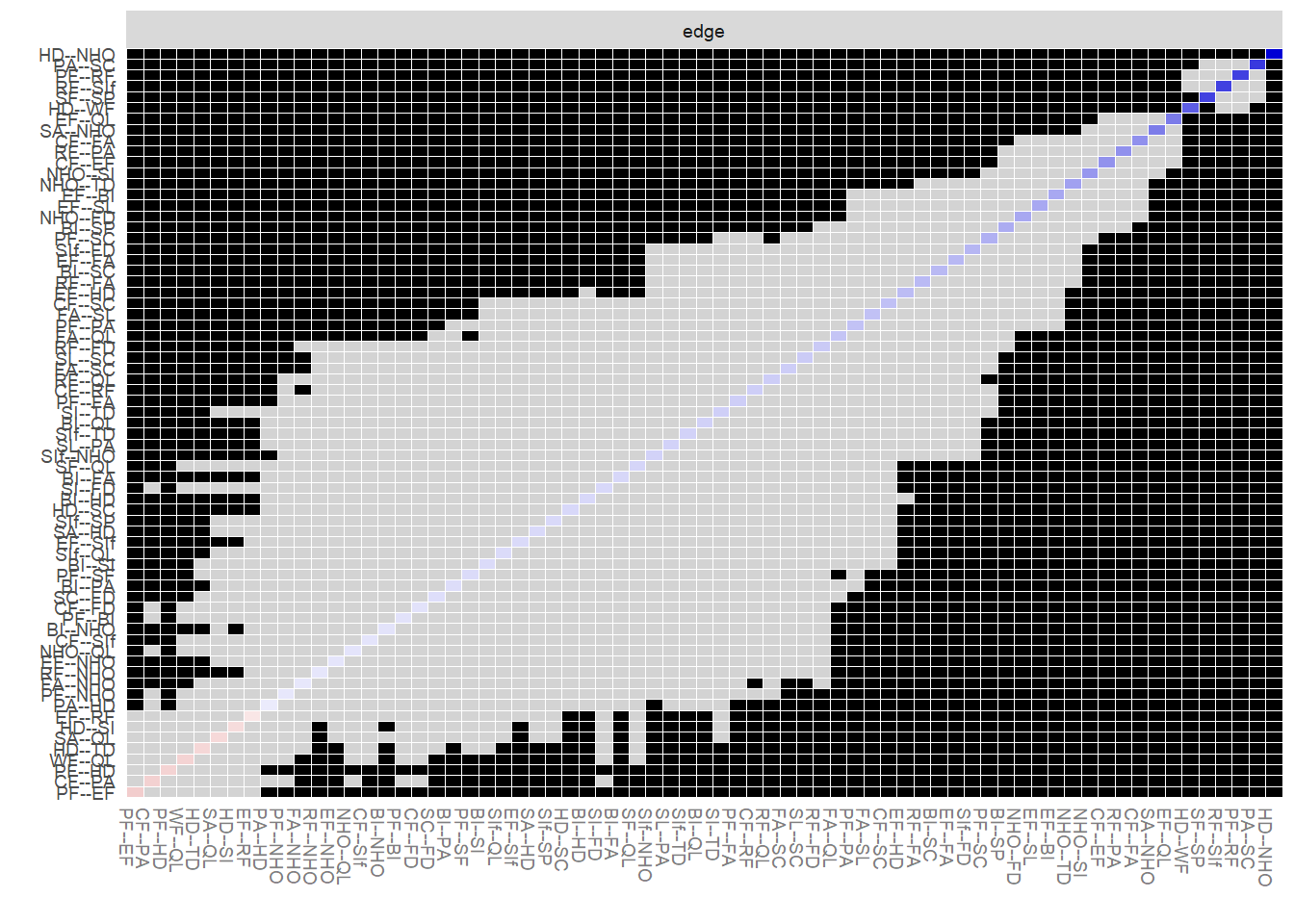
**Supplementary material Figure S1. Edge-weight difference plot** – This plot shows if the differences in edges of our partial correlation network model are significant. A black cell represents a significant difference between two edges, while a grey cell represents a non-significant difference. Colored boxes in the plot correspond to the color of the edge in Figure 1.


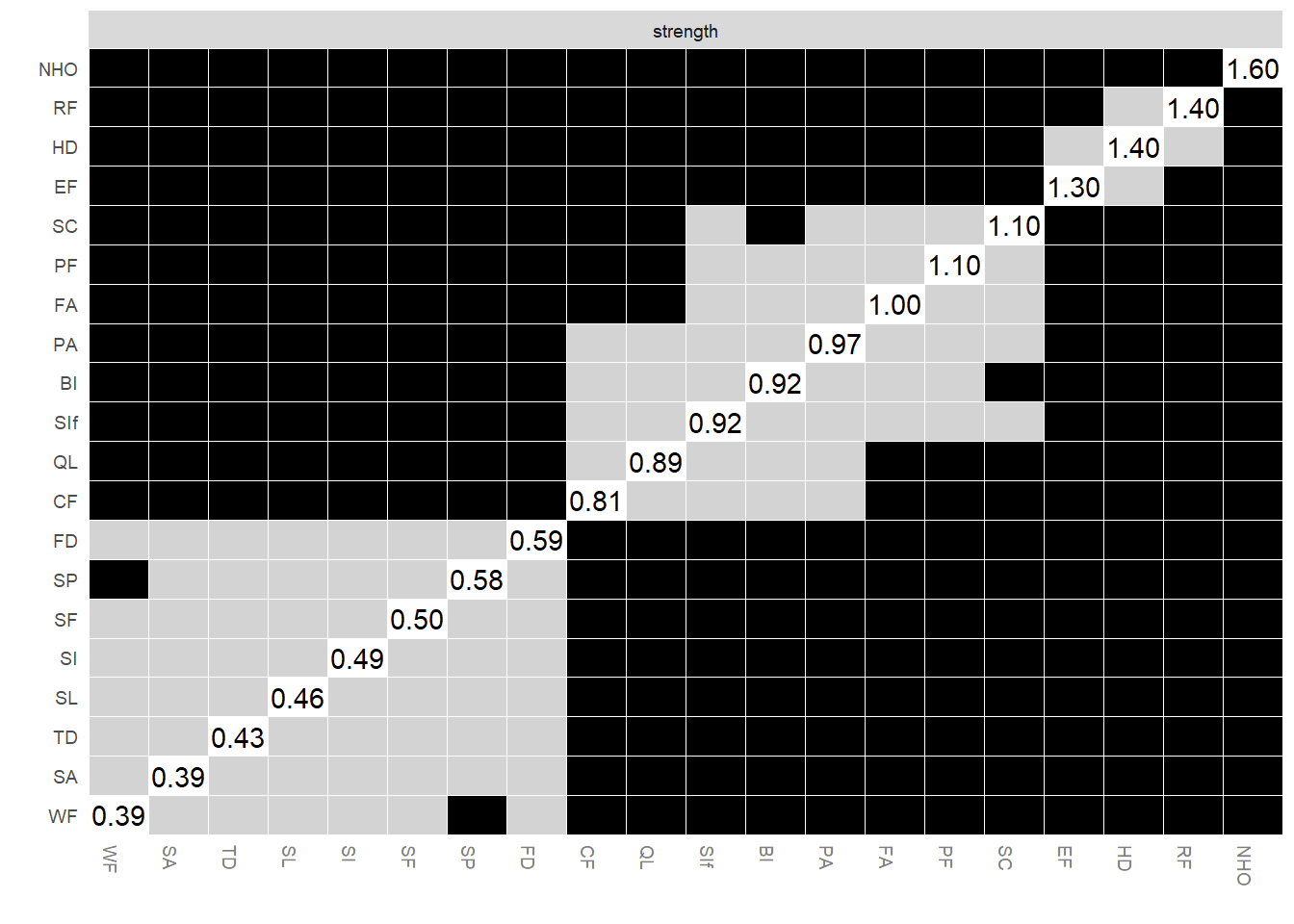


**Supplementary material Figure S2. Centrality difference plot** – This plots shows the comparison between the strength of each node. A black cell represents a significant difference between two the strength of two nodes, while a grey cell represents a non-significant difference. The values in white boxes represent the strength value of each node.
